# Biomarkers in Pediatric ARDS: Future Directions

**DOI:** 10.3389/fped.2016.00055

**Published:** 2016-06-01

**Authors:** Benjamin E. Orwoll, Anil Sapru

**Affiliations:** ^1^Department of Pediatrics, Division of Critical Care, University of California San Francisco, San Francisco, CA, USA; ^2^Department of Pediatrics, Division of Critical Care, University of California Los Angeles, Los Angeles, CA, USA

**Keywords:** biomarkers, acute lung injury, ARDS, PARDS, pediatrics, critical care, surrogate outcomes, molecular phenotypes

## Abstract

Acute respiratory distress syndrome (ARDS) is common among mechanically ventilated children and accompanies up to 30% of all pediatric intensive care unit deaths. Though ARDS diagnosis is based on clinical criteria, biological markers of acute lung damage have been extensively studied in adults and children. Biomarkers of inflammation, alveolar epithelial and capillary endothelial disruption, disordered coagulation, and associated derangements measured in the circulation and other body fluids, such as bronchoalveolar lavage, have improved our understanding of pathobiology of ARDS. The biochemical signature of ARDS has been increasingly well described in adult populations, and this has led to the identification of molecular phenotypes to augment clinical classifications. However, there is a paucity of data from pediatric ARDS (pARDS) patients. Biomarkers and molecular phenotypes have the potential to identify patients at high risk of poor outcomes, and perhaps inform the development of targeted therapies for specific groups of patients. Additionally, because of the lower incidence of and mortality from ARDS in pediatric patients relative to adults and lack of robust clinical predictors of outcome, there is an ongoing interest in biological markers as surrogate outcome measures. The recent definition of pARDS provides additional impetus for the measurement of established and novel biomarkers in future pediatric studies in order to further characterize this disease process. This chapter will review the currently available literature and discuss potential future directions for investigation into biomarkers in ARDS among children.

## Introduction

Acute lung disease or injury is a frequent contributor to admission to pediatric (PICU) and/or neonatal (NICU) intensive care units. At least 30% of children require invasive mechanical ventilation, and those who develop the acute respiratory distress syndrome (ARDS) may account for as much as 30% of all PICU mortality (1, 2). As in adults, acute lung injury (ALI) in children occurs as a consequence of various direct (e.g., infectious pneumonia, bronchiolitis, aspiration, traumatic lung contusion) or indirect (e.g., sepsis, shock, massive blood transfusion, non-pulmonary trauma) injuries to the lung. Similar events can lead to the infant respiratory distress syndrome (RDS), with surfactant deficiency and lung immaturity as underlying conditions (3). ARDS by the most recent Berlin definition (4), formerly known as the severe subgroup of “acute lung injury,” can result from these processes (5). Though a new pediatric-specific consensus definition for pediatric ARDS (pARDS) has been published (6), the vast majority of available studies in children are based on prior definitions. Clinically, ARDS is characterized by non-cardiogenic pulmonary edema, resulting in hypoxemia and infiltrates on chest radiography (7). Histopathologically, ARDS is characterized by a dysregulated lung inflammation and protein-rich interstitial and alveolar edema, as a consequence of enhanced permeability or disruption of the alveolar–capillary barrier (7). Disruption of the alveolar–capillary barrier can thereby lead to protein leak and transmigration of soluble proteins and other biological mediators both from and to the systemic circulation (8).

There has been growing literature resulting from interest in studying biological markers of acute lung diseases, including ARDS, in both the plasma or serum as well as bronchoalveolar lavage (BAL) fluid, with the hope to better elucidate pathophysiological mechanisms of ALI and to identify the potential markers of severity and outcome (6). The vast majority of these investigations have involved adult patients, and findings have subsequently been extrapolated to children. Large pediatric studies have been infrequent and limited in their findings because of the small numbers of ARDS patients, heterogeneity of underlying disease processes leading to pARDS, and the overall low mortality of pARDS. Multiple clinical outcome predictors in pARDS have been identified, with findings indicating that the severity of oxygenation defects, dead space fraction, organ failures, and underlying immunodeficiency are important factors (2, 9–11). However, the utility of clinical predictors is limited by the relatively low and decreasing rates of mortality in pARDS (12). Additionally, some clinical predictors may have less specificity for ARDS than molecular markers and have not yet led to the development of novel therapies targeting specific patient groups. Therefore, in addition to clinical findings there has been an ongoing interest in using biomarkers, specifically of endothelial and epithelial integrity/injury, coagulation/fibrinolysis, inflammation, and inflammation-associated cellular derangements, as surrogate markers of patient outcomes in pARDS.

The main focus of this review is to assess the current knowledge of biomarker patterns in acute lung disease/injury and their association to patient outcomes, with a specific focus on the pediatric and neonatal patient population.

## Biomarker Subgroups

Proteins and other biomarkers measured in blood or BAL fluid that have been reported in the literature and have the potential to serve as biological markers are presented here in a systematic manner based on their pathogenesis: inflammation, coagulation, epithelial, endothelial, surfactant, and non-protein markers. Plasma or serum biomarkers are favored in many instances over biomarkers from BAL fluid due to the relatively easy availability of blood samples. Additionally, in many instances, unstable patients with lung injury may not tolerate the BAL procedure. Plasma and serum biomarkers that have been studied in the pediatric or neonatal populations are presented in Table 1 and BAL fluid markers in Table 2. Most biomarkers studied to date are protein-based, but we will also discuss several non-protein biomarker groups that have the potential to add to our understanding of pARDS.

**Table 1:** **Serum/plasma biomarkers**.

Biomarker	Full name	Finding in children	Reference
**Inflammation**			
CRP	C-reactive protein	– Elevated in children with ARDS and associated with mortality and fewer VFDs	(13)
GM-CSF	Granulocyte–Macrophage colony-stimulating factor	– Elevated in severe influenza non-survivors (27% had ARDS)	(14)
HNE	Human neutrophil elastase	– Elevated in RSV bronchiolitis compared with controls, not correlated with disease severity	(15)
IL-1	Interleukin 1	– Elevated in SARS patients relative to control	(16)
IL-4	Interleukin 4	– Early elevation associated with ARDS and duration of ventilation in burn patients	(17)
IL-6	Interleukin 6	– Elevated in children with ARDS compared to control – Elevated in severe influenza non-survivors (27% had ARDS) – Decreased with steroid administration in ARDS trial and in SARS patients – Increased in burn patients with inhalation injury who did not survive, associated with ARDS, ventilation	(14, 16–20)
IL-7	Interleukin 7	– Decreased in burn patients with inhalation injury who did not survive, associated with ARDS, ventilation	(17)
IL-8	Interleukin 8	– Elevated in severe influenza non-survivors (27% had ARDS) – Elevated in BAL and plasma in ARDS vs. control	(14, 21)
IL-10	Interleukin 10	– Elevated in newborns with RDS independent of gestational age – Increased in burn patients with inhalation injury who did not survive, associated with ARDS, ventilation – Associated with O_2_ use at PICU discharge and plateau pressure in ARDS	(17, 20, 22)
IL-12	Interleukin 12	– Decreased in newborns with RDS independent of gestational age – Negative predictor of plateau pressure at ARDS day 7	(20, 22)
IL-13	Interleukin 13	– Increased in burn patients with inhalation injury who did not survive, associated with ARDS, ventilation	(17)
IL-17	Interleukin 17	– Elevations associated with O_2_ requirement at ICU discharge in children with ARDS	(20)
IP-10	IFN-inducible protein 10 (aka CXCL10)	– Elevated in severe influenza non-survivors (27% had ARDS)	(14)
sL-selectin	Soluble L-selectin	– Decreased in infants with RDS who developed BPD, increases with steroid therapy	(23)
MCP-1	Monocyte chemotactic protein 1	– Elevated in severe influenza non-survivors (27% had ARDS)	(14)
MIP-1alpha	Macrophage inflammatory protein 1 alpha	– Elevated in severe influenza non-survivors (27% had ARDS)	(14)
TNF-α	Tumor necrosis factor alpha	– Associated with IL-1 and IL-10 levels in children with ARDS	(20)
**Coagulation and fibrinolysis**			
AT-III	Antithrombin-III	– Decreased to <70% in non-survivors among pediatric ARDS patients	(24)
PAI-1	Plasminogen activator inhibitor 1	– Associated with mortality and fewer VFDs in children with ARDS – Significantly elevated in children with ARDS, septic shock, and purpura	(25, 26)
suPAR	Soluble urokinase-type plasminogen activator receptor	– Elevated in newborns who progress to BPD and associated with disease severity – Elevated in children with pneumonia and associated with severity	(27, 28)
**Epithelium**			
CC16/CC10	Clara cell secretory protein	– Elevated in ventilated preterm neonates – Cord blood levels decreased in RDS and BPD – Decreased in newborns who develop BPD	(19, 29, 30)
sICAM-1	Soluble intercellular adhesion molecule 1	– Elevated in children with ALI – Associated with mortality and length of ventilation in children with ARDS – Increases after lung recruitment maneuver are associated with non-response in children with ARDS	(31–34)
KL-6	Krebs von den Lungen-6	– Elevated in PICU patients with ARDS compared with sepsis/trauma and associated with mortality and LOS – Elevated in infants with RSV bronchiolitis and associated with disease severity	(35, 36)
sRAGE	Soluble receptor for advanced glycation end products	– Elevated in infants with bronchiolitis – Elevated in infants with RDS – Elevation predicts ALI in post-op cardiac surgery patients and associated with prolonged ventilation	(37–39)
**Endothelium**			
Ang-2	Angiopoietin-2	– Elevations associated with mortality in children with ARDS. Increases predict ARDS mortality in bone marrow transplant	(40)
sE-selectin	Soluble E-selectin	– Elevated in children with ALI compared with controls and associated with length of ventilation and mortality – Elevated in cord blood and plasma from infants with RDS who developed BPD – Decreases in response to steroid therapy in infants with RDS	(23, 41–43)
ET-1	Endothelin 1	– Elevated in cord blood from infants with RDS and correlated with severity – Elevated in children with ARDS and associated with mortality	(18, 44)
sTM	Soluble thrombomodulin	– Elevated and associated with mortality in children with indirect ARDS – Associated with organ failure in all children with ARDS	(45)
VEGF	Vascular endothelial growth factor	– Decreased in premature infants with RDS, also decreased VEGF to sVEGF-R ratio – Elevations associated with decreased pulmonary deterioration in premature infants	(46, 47)
sVEGF-R	Soluble vascular endothelial growth factor receptor	– Increased in premature infants with RDS	(46)
vWF	Von Willebrand factor	– Early elevation in pediatric ARDS associated with mortality and length of ventilation	(48)
**Surfactant proteins**			
SP-A	Surfactant protein A	– Elevated in children with ARDS	(21)
SP-B	Surfactant protein B	– Elevated in children with ARDS	(21)
SP-D	Surfactant protein D	– Elevated in children with ARDS – Elevated in children with bronchopneumonia – Elevated in infants with viral bronchiolitis	(21, 49–51)
**Other proteins**			
AQP-5	Aquaporin-5	– Elevated in neonatal RDS and correlated disease severity	(38)
BNP	B-type natriuretic peptide	– Elevated in non-surviving children with ALI – Elevations associated with prolonged ventilation, worse hypoxia, need for inotropes in children with ALI	(52)
sFAS-L	Soluble FAS ligand	– Elevated in infants with RSV bronchiolitis	(53)
sST2	Soluble suppression of tumorigenicity 2	– Elevated in newborns who progress to BPD	(27)
**Non-protein biomarkers**			
PGE	Prostaglandin E	– Elevated in RDS with decreased PGE to PGF ratio	(54)
PGF	Prostaglandin F	– Elevated in RDS with decreased PGE to PGF ratio	(54)

*ALI, acute lung injury; ARDS, acute respiratory distress syndrome; BPD, bronchopulmonary dysplasia; CLD, chronic lung disease; LOS, length of stay; O_2_, oxygen; RDS, respiratory distress syndrome; SARS, severe acute respiratory syndrome; VFD, ventilator-free days*.

**Table 2:** **Biomarkers in BAL fluid**.

Biomarker	Full name	Finding in children	Reference
**Inflammation**			
HNE	Human neutrophil elastase	– Elevated in nasal secretions of infants with RSV bronchiolitis – Non-significant elevation in children with ARDS compared to control	(15, 55)
IL-1	Interleukin 1	– Elevated in infants with RDS and associated with progression to CLD	(56, 57)
IL-6	Interleukin 6	– Elevated in healthy neonates subjected to mechanical ventilation – Elevated in infants with RDS and associated with progression to CLD	(56, 58–60)
IL-8	Interleukin 8	– Elevated in intubated children with acute lung injury – Elevated in preterm infants who progress to CLD	(21, 58)
sL-selectin	Soluble L-selectin	– Elevated in infants with RDS who develop BPD	(61)
MMP-8	Matrix metalloproteinase 8	– Elevated in children with ARDS – Associated with prolonged ventilation in children with ARDS – Elevated in infants with RDS and associated with progression to CLD	(55, 62–64)
MMP-9	Matrix metalloproteinase 9	– Elevated in children with ARDS – Associated with prolonged ventilation in children with ARDS – Elevated in infants with RDS and associated with progression to CLD	(55, 62, 64)
MPO	Myeloperoxidase	– Elevated in children with ARDS	(55)
TIMP-1	Tissue inhibitor of metalloproteinases 1	– Elevated MMP to TIMP ratios associated with prolonged ventilation in children with ARDS	(64)
TIMP-2	Tissue inhibitor of metalloproteinases 2	– Decreased levels in infants with RDS, associated with worse oxygenation and prolonged mechanical ventilation	(62)
TNF-α	Tumor necrosis factor alpha	– Elevated in healthy neonates subjected to mechanical ventilation	(60)
**Coagulation and fibrinolysis**			
PAI-1	Plasminogen activator inhibitor 1	– Elevated in ventilated pediatric patients who develop VAP compared with bacterial colonization	(65)
**Epithelium**			
CC10/CC16	Clara cell secretory protein	– Correlated with gestational age and with pulmonary infection in neonates	(66)
sICAM-1	Soluble intercellular adhesion molecule 1	– Elevated in infants with RDS and associated with progression to CLD	(67)
**Surfactant proteins**			
SP-A	Surfactant protein A	– Decreased in children with ARDS – Decreased in chronic aspiration pneumonitis – Decreased in severe RSV bronchiolitis	(68–71)
SP-B	Surfactant protein B	– Decreased in children with ARDS – Decreased in severe RSV bronchiolitis	(68, 69)
SP-D	Surfactant protein D	– Elevated in intubated children with acute lung injury – Increased in children with ventilator-associated pneumonia – Decreased in chronic aspiration pneumonitis – Decreased in severe RSV bronchiolitis	(21, 69, 71, 72)
**Non-protein biomarkers**			
PGI_2_	Prostacyclin	– Elevations in infants with RDS associated with decreased oxygen need and decreased length of ventilation	(73)
TXB_4_	Thromboxane B_4_	– Elevated in ventilated infants who progress to BPD	(57)

*ARDS, acute respiratory distress syndrome; BPD, bronchopulmonary dysplasia; CLD, chronic lung disease; RDS, respiratory distress syndrome; RSV, respiratory syncytial virus; VAP, ventilator-associated pneumonia*.

### Inflammation Pathways

Some of the most intensely studied protein biomarkers of critical illness belong to the large family of inflammatory cytokines dubbed interleukins (ILs). Many of the members of this family have been studied in other forms of critical illnesses, some of which are recognized ARDS risk factors, such as sepsis, trauma, or postoperative states. IL-1 and its antagonist IL-1 receptor antagonist (IL-1ra) have been found in BAL fluid and correlate with ARDS severity and outcome in adults (74). In children, IL-1 has not been correlated with ARDS, though it significantly elevated among a cohort of children with severe acute respiratory syndrome (SARS) (16). IL-6 is another important inflammatory cytokine, and elevated plasma and BAL fluid levels have been associated with ARDS in adults (74–77) as well as plasma in children and neonates with RDS (14, 18, 19). In studies of children with burn-related inhalation injury and severe influenza, IL-6 levels were associated with increased mortality (14, 17), in agreement with adult data (78). IL-1 and IL-6 levels in BAL fluid were also both found to be elevated in premature babies with RDS and were correlated with the development of chronic lung disease (CLD) (56, 58, 59). As a biomarker of response to therapy, IL-6 was recently found to decrease in response to glucocorticoid (GC) therapy in a randomized trial of GC therapy in children (20). The relationship of the inflammatory cytokine response to ventilation strategies was also demonstrated in the landmark ARDSNet trial of low-tidal-volume ventilation, where IL-6 and IL-8 levels were observed to decrease in the low-tidal-volume group compared with the controls (79, 80). IL-8, another inflammatory cytokine, is elevated in the plasma and BAL fluid of adults with ARDS and is associated with organ failure and mortality in adults (75, 77, 81). In children with ARDS and RDS, the plasma and BAL fluid IL-8 levels are elevated (21, 58), but the relationship with outcomes remains less clear. However, in a cohort that included critically ill children with influenza and 27% ARDS prevalence, non-survivors had dramatically higher plasma IL-8 concentrations than survivors, suggesting a possible role as a prognostic factor in pARDS more generally (14). IL-10 is a major anti-inflammatory cytokine, and elevations in plasma and BAL fluid have been associated with the development of ARDS as well as with ARDS mortality in adults (80, 82, 83). It appears that an early rise in IL-10 at the onset of ARDS is followed by normalization of levels over the first 21 days of illness (74), and this pattern was also seen in children with ARDS, both with and without steroid therapy (16, 20). IL-10 is elevated in newborns with RDS (22), independent of gestational age, and has also been associated with increased development of ARDS and increased mortality among pediatric burn patients with inhalation injury (17). Several other cytokines of the IL family, including IL-4, IL-7, and IL-13 (each in pediatric burn patients) (17), IL-12 (RDS) (22), and IL-17 (pARDS) (20), have been associated with the outcomes from lung injury in individual studies. Comparing non-survivors to survivors from the previously mentioned cohort of severe influenza patients, the investigators also found significant elevations in granulocyte–macrophage colony-stimulating factor, interferon-inducible protein 10, monocyte chemotactic protein 1, and macrophage inflammatory protein 1α (14), though these findings have yet to be reproduced in other pediatric cohorts.

Tumor necrosis factor alpha (TNF-α) and its soluble receptors (sTNFR-1 and sTNFR-2) have diverse effects on the immune system and have been associated with both ARDS development and outcomes in adults (74, 78). The role of TNF-related activity in pARDS is less clear. A study of healthy neonates subjected to mechanical ventilation found increases in TNF-α and IL-6 in tracheal aspirate fluid (60) and plasma TNF-α levels were correlated with other cytokine levels, including IL-1α and IL-10 among children in a randomized controlled trial of corticosteroids for ARDS (20), suggesting a possible role in pARDS.

Another group of inflammation-related proteins that has been of significant interest is the matrix metalloproteinase (MMP) family. Through their activity in the breakdown and remodeling of tissue matrices (84), these proteins may have direct functional significance in addition to their utility as biomarkers. MMPs can be produced by multiple cell types, including immune cells, fibroblasts, and epithelial cells, and many different subgroups of MMPs have been identified and classified based on their substrates (85). Several studies have found associations between levels of MMP-8, MMP-9, MMP-10, and the MMP antagonist tissue inhibitor of metalloproteinases 1 (TIMP-1) and outcomes from sepsis in adults (86–88), while one study in a mixed population of ventilated adults found that serum levels of TIMP-1 and MMP-8 were associated with decreased survival and increased severity of hypoxemia (89). In pediatric patients, there is more substantial evidence base for the role of the MMPs in lung injury and ARDS. In 2001, two different groups demonstrated elevated levels of MMP-8 in the BAL fluid from preterm babies with RDS (62, 63) and found that higher amounts of MMP-8 are associated with progression from RDS to CLD or bronchoalveolar dysplasia (BPD). One group also identified an imbalance between MMP-8 and its inhibitor, TIMP-1, in the BAL fluid, and lower levels of TIMP-1 were associated with hypoxemia and prolonged ventilation (62). In pARDS patients, two more recent reports have described elevations in BAL fluid MMP-8 and MMP-9 levels in ARDS patients relative to controls as well as associations between these two proteins and the need for prolonged mechanical ventilation (55, 64). One study also examined the relationship between these MMPs and other neutrophil-derived proteinases, such as human neutrophil elastase (HNE) and myeloperoxidase (MPO), finding positive relationships that may indicate neutrophil activity as a major source for the MMP elevations seen in ARDS (55).

C-reactive protein (CRP), which is typically highly elevated in and used as a marker for acute inflammatory states, has been found to be inversely associated with mortality in an adult ARDS population (90). However, in a subsequent study of 98 ventilated pediatric patients with ALI the investigators found that higher CRP levels drawn within 48 h of ALI diagnosis were associated with mortality and fewer ventilator-free days (VFDs) (13). These discordant findings must be interpreted in the context of the multiple potential confounding influences in the two studies, but also serve to highlight the level of variability seen in the results of biomarker studies and promote a cautious approach in the implementation of clinical decisions based on them.

Though many inflammatory cytokines have been studied in pARDS, no single one has demonstrated clear superiority over others as a single marker for the disease process. IL-6 has been most intensely studied, but perhaps a combination of cytokines may offer a more complete picture of the inflammatory processes active in pARDS. Many of these molecules also have specific inhibitors, offering enticing prospects for new treatment modalities.

### Coagulation/Fibrinolysis-Related Biomarkers

In the classic pathological description of ARDS, the alveolar spaces are filled with a proteinaceous complex that forms the characteristic hyaline membranes (7). This complex is composed of, in large part, fibrin and fibrin breakdown products in addition to pulmonary edema fluid and inflammatory infiltrates. This suggests a role for disordered coagulation homeostasis in ARDS and related conditions, and preclinical models support the role of pulmonary coagulopathy as a pathologic mechanism in ALI (91). Derangements in typical measures of function of the coagulation system, such as the prothrombin time (PT) and activated thromboplastin time (aPTT), have been associated with increased mortality and organ failure in children with ARDS (92). Platelets, also critical to the function of the coagulation system, are dysregulated in ARDS (93, 94), and thrombocytopenia at ARDS onset has been reported to correlate with increased morbidity and mortality (95) in both adults and children (96).

However, in addition to these familiar markers of coagulation, multiple, more novel biomarkers have been evaluated in children with ARDS. Plasminogen activator inhibitor 1 (PAI-1) is an antifibrinolytic enzyme that is activated in the setting of inflammation. Depressed fibrinolytic activity, demonstrated by elevated PAI-1 levels in the BAL fluid and blood from adults with ARDS, is associated with increased mortality and decreased VFDs (97–99). PAI-1 has also been evaluated as a marker in pediatric patients, and among a cohort of pediatric patients with ARDS, elevated plasma PAI-1 was also associated with increased mortality and decreased VFDs (25). Among cohorts of critically ill children, PAI-1 levels from BAL fluid were also able to discriminate patients with ventilator-associated pneumonia (VAP) compared with colonized patients (65), and plasma levels were significantly elevated in patients with septic shock, purpura, and ARDS (26). In addition to PAI-1, another important regulator of coagulation activity in lung disease is soluble urokinase-type plasminogen activator receptor (suPAR). This protein binds urokinase (uPA) at the cell surface and facilitates the further proteolytic activation of plasminogen and pro-uPA, while the soluble form may have additional roles in inflammation (100). Plasma suPAR levels were elevated in a large study of critically ill, ventilated adults and were associated with mortality (101). In a neonatal study, blood suPAR levels were elevated among those who developed BPD compared with controls, and correlated with disease severity (27). Similarly, suPAR levels were shown to correlate with severity in a cohort of children with pneumonia, though none of that cohort developed ARDS (28). Decreased plasma levels of antithrombin-III, which mediates heparin’s anticoagulant effect, early in the course of ARDS have also been associated with increased mortality in both adults and children (24, 102). Though protein C, an important regulator of the activity of thrombin, has been investigated as both a biomarker (103) and as a therapeutic agent in the form of activated protein C (APC) (104–106) in adult ARDS patients, additional studies are needed to clarify its role in children with ARDS. Further investigation into biomarkers of coagulation and fibrinolysis pathways, including the roles of tissue factor (TF) and its pathway inhibitor (TFPI), may further advance our understanding of the pathogenesis of ARDS in children and provide for new therapeutic targets.

Disturbances of coagulation and fibrinolysis in the setting of pARDS are important in the pathogenesis of the disease, especially as they relate to the function of the microcirculation and development of organ failure. PAI-1 and the suPAR pathways have the most evidence in children, but more dynamic and complete understanding of coagulation pathways will be critical to improving outcomes in children.

### Epithelium-Related Biomarkers

Pulmonary alveolar and bronchial epithelium is an integral component of the alveolar–capillary barrier, which is disrupted in ARDS. It may be the site of the primary injury, as in direct lung injury-mediated ARDS, or may become injured secondarily as a result of indirect mechanisms of lung injury (5). Markers of epithelial cell injury have been used both as indicators of the severity of lung injury as well as for the purpose of differentiation between patients with different mechanisms of injury.

A glycoprotein that has been highly associated with epithelial cell damage and increased permeability is Krebs von den Lungen-6 (KL-6) (107). KL-6 is present on alveolar type II pneumocytes and can be released in the serum or the alveolar lining fluid (108). Elevated KL-6 levels in the blood and in BAL fluid have been associated with the development of ARDS (109–111) as well as worse ARDS outcomes in adult patients (109, 112, 113). In a study of children with ARDS compared with sepsis or traumatic brain injury, serum KL-6 was elevated in ARDS patients and higher levels were associated with increased mortality (35). In that study, KL-6 levels were also correlated with measures of oxygenation, length of ventilation, and LOS. Other pediatric studies have demonstrated elevated serum KL-6 in infants with bronchiolitis (36) or BPD (29, 114), as well as with respiratory complications in severely developmentally disabled children (115) and in pediatric interstitial lung disease (116).

Clara (also known as club) cell secretory protein (CC16) is a protein that is produced and secreted by the Clara cells of the tracheobronchial tree and has been identified as a marker of respiratory epithelial injury (117). Elevations in plasma levels of CC16 in adult patients have been associated with both the development of ARDS (118) as well as with increased mortality and organ failure scores when compared with controls (119, 120). However, when Ware et al. compared CC16 levels between patients with ALI and those with cardiogenic pulmonary edema they found that the ALI patients had comparatively lower BAL fluid and plasma CC16 levels, suggesting that CC16 elevations may not be specific to ARDS (121). In children, CC16 has been studied primarily as a biomarker in neonatal RDS and for the development of BPD. However, the results are conflicting. Reports from cord blood and postnatal serum found lower levels of CC16 in patients who went on to develop BPD (19, 29), while one study demonstrated elevated postnatal serum levels in mechanically ventilated infants and correlations with progression to BPD (30). Yet, another study of tracheal aspirates in preterm infants showed instead a correlation with gestational age as well as elevations in the setting of acute infection (66). Though there is significant interest in CC16 as both a biomarker and as a potential therapy (122), the evidence continues to be inconsistent and more study in larger populations is needed.

Soluble receptor for advanced glycation end products (sRAGE) is a protein that is preferentially expressed on the basolateral membrane of alveolar type 1 cells, which are the predominant epithelial surface cells in the lung (123). Adults’ studies have shown elevations in plasma and BAL fluid sRAGE in ARDS (124), and elevated plasma sRAGE is correlated with increased mortality, organ failure, and reduced alveolar fluid clearance (125, 126). Pediatric studies have demonstrated increased plasma sRAGE in patients with bronchiolitis and with RDS (37, 38), and plasma sRAGE from pediatric postoperative cardiac surgery patients were associated with development of ALI as well as with worse oxygenation and longer periods of mechanical ventilation (39). It has been noted that BAL fluid sRAGE levels may vary inversely with age, which should be accounted for in future studies, though no such variations have been reported with plasma sRAGE (127).

The alveolar–capillary barrier is also maintained in part by cellular adhesion molecules, and one of these proteins that has become established as an important marker of both epithelial and endothelial integrity is soluble intercellular adhesion molecule 1 (sICAM-1) (128–131). In adults, there are multiple studies associating blood and BAL fluid sICAM-1 levels with the development of ARDS as well as with ARDS outcomes, such as mortality (132–134). These findings have been confirmed in studies of children, first when Flori et al. described increased mortality in children with ARDS, which was independently associated with elevations of sICAM early in the disease course, especially at levels >1000 ng/mL (31). This was reproduced in children with ARDS receiving high frequency oscillating ventilation, and further elevations were seen in patients who did not respond to lung recruitment maneuvers (32, 33). A recent study also demonstrated early increases in sICAM-1 levels in children with ALI compared with controls and found associations between those levels and both durations of ventilation and mortality (34). sICAM has been studied in tracheal aspirates of infants with RDS, and concentrations were significantly higher in those who went on to develop CLD (67). These findings are highly consistent and underscore the importance of adhesion molecules and the pulmonary alveolar–capillary barrier in the pathogenesis of pediatric lung injury.

Markers related to the integrity of the pulmonary epithelium have strong associations with outcomes and measures of disease severity in pARDS. KL-6, CC16, sRAGE, and sICAM are examples of proteins specific to the epithelium that have shown promise as biomarkers and which may prove useful in further characterizing subpopulations of children with more severe epithelial injury.

### Endothelium-Related Biomarkers

The endothelium represents the internal side of the alveolar–capillary barrier and has been studied extensively as a mediator of multiple disease states, including sepsis and ARDS. Pulmonary endothelial cells (ECs) are at risk of injury from multiple potential sources, both transmitted from external sources through the pulmonary epithelium as well as from internal sources through the systemic circulation. Endothelial damage and dysfunction are thought to be a major mechanism for the development of the multi-organ dysfunction that is characteristic of severe ARDS. The endothelium has multiple active roles, including production of adhesion molecules and cytokines, modulation of vascular tone, and management of intravascular coagulation (135).

Several protein biomarkers have been studied as surrogate markers for endothelial injury or increased permeability. Von Willebrand factor (vWF), a protein involved in platelet function produced by ECs and released into the blood upon their activation or damage, was elevated in the plasma of children with ARDS, and early elevation was correlated with mortality and decreased VFDs (48). These results were consistent with previous findings in infants with RDS (136) as well as with multiple adult studies (137–139). Soluble E-selectin is an endothelial leukocyte adhesion protein that is upregulated under conditions of inflammation (140). E-selectin levels have been associated with ARDS in adults (130, 141, 142), and a recent study reported elevated plasma E-selectin levels in children with ARDS compared with controls as well as an association with mortality (41). Among infants with RDS, plasma E-selectin levels have been associated with the development of BPD and were found to decrease with dexamethasone therapy (23, 42, 43). L-selectin is another member of the selectin family of leukocyte adhesion molecules and is expressed primarily on leukocytes (140). Consistent with studies in adults (102, 143, 144), L-selectin levels in the plasma of infants with RDS are decreased among those patients who develop BPD (23) while levels in the BAL fluid are increased, suggesting possible transmigration of leukocytes into the alveolar space (61). Vascular endothelial growth factor (VEGF), which promotes endothelial and vascular growth, has also been evaluated in neonates with RDS and was found in decreased concentrations in patients with severe RDS, while its soluble receptor (sVEGF-R) was increased (46). No correlations between BAL fluid VEGF levels and disease incidence or severity have been identified in studies of infants with RDS or BPD (46, 145).

Another protein that is produced by the pulmonary endothelium as well as other cell types, including epithelium, smooth muscle, and immune cells, is endothelin 1 (ET-1). It has garnered interest in ARDS investigations due to pleiotropic effects, including potent pulmonary vasoconstriction and stimulation of inflammatory responses (146). ET-1 levels were examined in two pediatric studies, one in neonates and one in children. The neonatal group showed higher cord blood ET-1 levels in infants with RDS than controls, and ET-1 levels correlated with longer duration of mechanical ventilation (44). The study in children with ARDS demonstrated early elevations in ET-1 in affected children compared with controls and also found a significant association between ET-1 levels in the first 24 h of disease and survival (18). These findings are consistent with limited data on plasma (147) and BAL (148) ET-1 levels in adult ARDS patients, and further research may help guide future investigations of potential ET antagonist or inhaled nitric oxide therapies for ARDS patients.

Soluble thrombomodulin (sTM) is a circulating protein formed as a result of proteolytic cleavage of cellular thrombomodulin, a membrane bound protein involved in coagulation and inflammatory pathways, and has been identified as a marker of endothelial injury (149, 150). Our group has found significant relationships between early elevations in plasma sTM and organ failure in a large pARDS cohort, and sTM was predictive of mortality in a subset of those patients with indirect mechanisms of lung injury (45). These findings were consistent with previous results from our group in adult patients with ARDS (151) and may hint at potential opportunities for endothelial functional replacement through the use of recombinant thrombomodulin, which has been increasingly used in cases of sepsis and critical coagulopathies (152–154).

As a mediator of vascular permeability, angiopoeitin-2 (Ang-2) may play a significant role in the development of and outcomes from ARDS (155). In adults with ARDS, Ang-2 levels in the plasma are elevated compared with controls and predict mortality in medical and trauma-related ARDS (83, 156–158). Though data remain relatively sparse among pediatric patients, we recently reported that Ang-2 elevations are associated with mortality in pARDS and, additionally, that rising Ang-2 levels are highly predictive of mortality among hematopoietic stem cell transplant recipients (40).

The endothelium, with its diverse functions involving, among others, inflammation, coagulation, and hemodynamics, has a central role in the pathobiology of ARDS in children. Markers, such as vWF, selectins, ET-1, sTM, and Ang-2, are associated with the endothelium in ARDS, each with a different functional implication. Clinical assessments of these markers will help construct a more complete view of the endothelial function and guide future therapeutic trials.

### Surfactant Proteins

The surfactant proteins are a group of proteins produced by the alveolar type II cells within the lung and secreted as part of a complex substance called pulmonary surfactant that serves primarily to reduce surface tension in the lung. Alterations or damage to the pulmonary surfactant system have been proposed as a mechanism for some of the changes in pulmonary physiology seen in ARDS (7, 159). Several studies have examined surfactant protein levels in pediatric patients with lung injury. One large study examining 120 children with respiratory failure requiring intubation found lower levels of tracheal aspirate surfactant protein A (SP-A) and surfactant protein B (SP-B) relative to total aspirate fluid protein in pneumonia and ARDS patients compared with controls. SP-A levels were also correlated with pulmonary compliance (68). Another, smaller study also found decreased normalized levels of SP-A and SP-B in BAL fluid from children with ARDS, while surfactant protein D (SP-D) was found in elevated amounts with increased breakdown products (21). The same study also evaluated plasma SP levels, finding that all three SPs (A, B, and D) were elevated in plasma at the time of ARDS diagnosis and that plasma SP-B was independently associated with LOS and duration of mechanical ventilation. Elevations in BAL fluid SP-D levels have also been found in children with VAP compared to ventilated controls (72), and plasma SP-D levels are also higher in children with bronchopneumonia and associated with increased need for supplemental oxygen (49). Similar findings of decreased levels of SP-A and SP-B in the BAL fluid were reported in studies of infants with severe bronchiolitis (69, 70) but, in contrast to other reports, SP-D was also reduced. However, SP-D in the serum was elevated in two studies of viral bronchiolitis (50, 51). In aggregate, these results seem to suggest a relative depletion of pulmonary surfactant proteins and a concomitant increase in plasma concentrations, which may relate to increased permeability at the alveolar–capillary barrier. Thus, plasma and BAL fluid surfactant concentrations have significant potential as biomarkers specific to intrapulmonary processes.

Though trials of surfactant replacement in pARDS have had mixed results (160), there are clearly disruptions to the normal surfactant system in this disease. Levels of specific surfactant proteins in the BAL fluid and blood are associated with clinical outcomes, and assessments of these markers in future trials could be used to stratify risk and may inform specific replacement therapies.

### Other Protein Biomarkers

Though the vast majority of biomarker data available in pediatrics fall into one of the above categories, several known markers do not fit easily into any of them. B-type (formerly known as brain-type) natriuretic peptide (BNP or pro-BNP) is a protein that has been commonly used to assess fluid status and cardiac strain in congestive heart failure and pulmonary hypertension, among others. Studies in adults have found that BNP can help discriminate ARDS from cardiogenic pulmonary edema, and higher BNP levels are associated with mortality (161–163). Similarly, in a cohort of children with ALI, elevated BNP levels early in the disease course were associated with worse oxygenation, fewer VFDs, increased need for inotropes, and were significantly elevated among non-survivors (52). These findings suggest BNP as a possible marker of the altered cardiopulmonary interactions that can occur in the setting of ALI in children.

Other markers with limited evidence in children with lung injury include soluble FAS ligand (sFAS-L) and caspase-1, which are involved in the process of programed cell death and were elevated among infants with RSV bronchiolitis compared with controls (53). Though there are some data to support a role for soluble FAS and sFAS-L in adult ARDS (164, 165), the utility of these two markers in pARDS remains unclear.

### Non-Protein Biomarkers

Though much of the investigation devoted to the identification of biomarkers in ARDS has been focused on proteins, a great deal of progress has also been made in the area of non-protein biomarkers. These non-protein markers are quite varied, but can be generally categorized into lipid-based molecules, nucleic acids, microparticles (MPs), and extracellular complexes. We will discuss each of these categories briefly.

Lipid biomarkers comprise a heterogeneous group of hydrophobic molecules that have multiple and varied roles in normal bodily functions. One group of these that has been investigated in more detail in the setting of ARDS is the eicosanoids, which are generated through metabolism of 20-carbon fatty acids, such as arachidonic acid. These include prostaglandins (PGs), leukotrienes (LTs), thromboxane (TXA), and others, many of which have potential effects involving vascular, endothelial, platelet, and leukocyte function. Thromboxane A_2_ (TXA_2_) and prostacyclin (PGI_2_) (also known as prostaglandin I_2_) are produced in the lung and have effects on pulmonary vascular resistance, with TXA_2_ promoting pulmonary vasoconstriction and PGI_2_ vasodilation (166). In several studies of at risk adults (167–169), TXA levels were elevated in patients who developed ARDS, as was the TXA to prostaglandin ratio. Indeed, elevations in BAL fluid TXA B_2_, an active metabolite of TXA_2_, are associated with progression to BPD in infants with lung inflammation and RDS (57), while elevations in PGI_2_ seem to have a beneficial relationship (73). An early study of RDS also reported elevations in plasma prostaglandin E (PGE) and prostaglandin F (PGF) with a low PGE:PGF ratio (54). Elevated levels of LT B_4_, C_4_, and D_4_ have been detected in the blood and BAL fluid from adult patients with ARDS (170, 171), and leukotriene B_4_ has been associated with mortality (81, 172). In children, these molecules have largely been studied in the setting of neonatal RDS and the development of BPD. Elevations of urinary leukotriene E_4_ have been associated with ARDS in adults (173) and development of BPD in infants (174, 175), though variations observed may be highly affected by gestational age and maturity (176). Several studies of neonates have demonstrated alterations in the levels of markers of oxidative stress and DNA damage in RDS (175, 177), and one study of BAL fluid in children with ALI found alterations in the concentrations of multiple cell surface lipids indicative of alveolar–capillary membrane damage (21). Eicosanoids and other lipid-derived and small molecules may serve as useful biomarkers, especially of vascular physiology, oxidative damage, and inflammation, in future studies of children with ARDS.

Advances in genetic science and technology have enabled research into the feasibility of using nucleic acid-based biomarker assays in acute illness. Major targets have been small RNA molecules, such as micro-RNAs (miRNAs), which, due to their abundance in the blood and other body fluids as well as their relative resistance to degradation, may have utility as biomarkers (178). miRNAs have a predictable structure and have variable expression patterns depending on the body site and physiologic conditions. miRNAs perform important functional roles in cellular function through their ability to regulate translation of specific gene products at the mRNA level, and a single miRNA species may have multiple gene targets (178). Another notable feature of miRNAs is that they have potential as therapeutic targets due to their ability to be regulated by antisense versions of themselves, or antagomiRs. Though many miRNAs have been identified as possible contributors to or markers of lung diseases, clinical data in humans with ARDS are extremely limited (179, 180). This is a fertile area for further study in children with ARDS.

Additional types of non-protein biomarkers that may have utility in pediatric lung disease include several types of extracellular complexes, including circulating MPs (also known as microvesicles or cellular exosomes) and neutrophil extracellular traps (NET). MPs are a class of cell-derived vesicles that develop by budding off from the membrane of many different cell types and range in size from 50 nm to 1 μm. They may carry a variety of different contents, including proteins, lipids, and nucleic acids, and have a wide variety of potential effects in lung injury depending on their origin, location, and contents (181). MPs have been isolated from the blood (182) and BAL fluid (183) in adult ARDS patients and have been subtyped based on their cellular origin with certain subtypes, such as leukocyte-derived MPs, demonstrating possible prognostic significance. MP-based biomarkers may prove useful in future studies of children with ARDS and may also have potential as therapeutic agents able to ferry payloads of drugs or other substances to specific target tissues based molecular tropism (184). NETs are derived from programed death of circulating neutrophils and are composed of extruded cellular contents, including nuclear chromatin, histone proteins, and other various proteins. These NETs have antimicrobial activity and have been proposed as a host defense mechanism (185). NETs have been detected in the plasma and BAL of patients with transfusion-related acute lung injury (TRALI) (186) and critical illnesses (187), and circulating extracellular histones, the major protein component of NETs, are directly cytotoxic and elevated in the setting of trauma-associated lung injury (188). NETs, MPs, and other extracellular complexes represent potentially useful biomarkers in children with ARDS.

## Discussion

Since the initial description of ARDS, there have been widespread investigations into the pathobiology of the syndrome as well as for better ways to care for its victims. Through the increasing use of molecular techniques for the study of the ARDS disease process, we have gained a great deal of knowledge related to the development, progression, and resolution of this devastating condition. One major hope for the application of this knowledge is to further characterize subpopulations within what is widely regarded as an extremely heterogeneous disease, which may then lead to the development of specific therapies tailored toward the pathophysiological changes found in each subpopulation. Preliminary steps in this process were realized recently with the description of a biomarker “panel” for ARDS (83) and the publication of a latent class analysis of two ARDS clinical trials by Calfee et al. who described two subphenotypes, hyper- and hypo-inflammatory, with differential clinical responses to the trial interventions (189). These findings were based on the analysis of clinical characteristics and multiple biomarkers and hold promise that further subphenotyping might be used to guide the design of future clinical trials, such as those targeting Th2 high vs. Th2 low populations in asthma (190). Though no clear ARDS subphenotypes have yet been identified in pediatric patients, it would seem that the same principles could reasonably be applied. It is also important to recognize that information from biomarkers in pARDS may provide value both in terms of their ability to improve risk stratification and also to better define the pathophysiology of this heterogeneous condition. Better risk stratification will allow for well-designed trials of pARDS therapies and improved prognostication for families. In counterpoint, improved understanding of the pathological mechanisms that lead to pARDS and regulate its resolution may form the bases for the development of novel therapies.

New types of biomarkers, such as non-protein and nucleic acid-based biomarkers, are potentially rich sources of information that remain relatively untapped in pediatric populations. Challenges remain, especially in the technical aspects of isolating, characterizing, and quantifying non-protein biomarkers in large numbers of children with ARDS. However, technological advancement, especially in the realm of nucleic acid sequencing with polymerase chain reaction (PCR)-based methods, has dramatically reduced both the price and processing time for assays of genetic material. Methods for large-scale isolation of lipid vesicle-based MPs are not yet well-developed but will certainly improve in the coming years. Both nucleic acids and lipid vesicles are very appealing as both markers and as potential therapeutic agents, as they have potential to act through tissue and biochemical pathway-specific mechanisms that may increase potency and decrease unwanted side effects. Non-protein biomarkers thus represent a significant field for future research in pARDS.

There are many remaining difficulties in the pursuit of accurate and useful biomarker-based models in pARDS. One major challenge has been the establishment and widespread use of common diagnostic criteria for ARDS in children. Though consensus criteria have been available for decades in adults (4, 5), a pediatric-specific definition for ARDS, now referred to as pARDS, was only published in 2015 (6). This definition expands upon the adult Berlin definition to allow for criteria that are more clinically relevant to pediatrics, such as unilateral radiographic infiltrates and pulse oximetry-based oxygenation metrics. It also utilizes the oxygenation index (OI), which has been well-validated in pediatric lung disease, as a risk stratification tool. This definition, as it becomes widely adopted, will help to improve the comparability between pediatric studies of ARDS, especially as the OI reflects not only the fraction of inspired oxygen but also the mean airway pressure support. Future studies of ARDS biomarkers in children should therefore strive to abide by these definitions, and indeed, many of the previously identified biomarkers of ARDS in children likely should be revalidated using the new pARDS criteria.

By definition, biomarkers are found and measured in body fluids and tissues. Therefore, to measure them, it is necessary to collect biological samples from some of our societies’ most vulnerable members. Children are more sensitive to collections of large volume samples than adults, and parents are often wary to allow their sick children to participate in clinical research. Fortunately, over recent years, many improvements in assay technology have allowed the development of multiplex assays that can measure dozens of proteins and other biomarkers in a single fluid sample, greatly improving the information yield from limited patient populations.

Technologies like these can help overcome a major challenge pARDS research, which is the overall paucity of data. However, this will also require a concerted effort toward collaboration between many researchers and institutions to expand the available pool of data. The systems of incentives in place for funding and promotion must also be reexamined to support collaborative research in addition to individual projects. Research groups, such as the Pediatric Acute Lung Injury and Sepsis Investigators^1^ and the Collaborative Pediatric Critical Care Research Network,^2^ among others, must continue to expand and share their databases of biomarker data to facilitate new insights and generate hypotheses for new clinical trials. Larger data sets may allow for improvements in sensitivity and specificity, as well as expand the external validity of, known biomarkers such that meaningful clinical tools or scores could be developed around them. However, in order to make use of these large data sets pediatric researchers will need to embrace the growing field of biomedical informatics. Large-scale information analysis can utilize complex algorithms and machine learning to efficiently detect patterns and subpopulations that would be otherwise invisible with conventional techniques. However, this requires dedicated resources and data access that is best suited to collaborative networks. No pARDS biomarker has yet achieved the clinical utility of creatinine for kidney injury or troponin for myocardial infarction, but with large-scale collaboration and data analysis the chance of success grows dramatically.

Data gleaned from biomarker testing may offer some of the most timely and valuable diagnostic and prognostic information available in the setting of ARDS. This is especially true in pediatric medicine, where invasive diagnostic procedures, such as bronchoscopy and biopsy, as well as radiation exposure related to diagnostic imaging, are limited as much as possible. In the setting of low mortality in pARDS, biomarkers or panels thereof may also be valuable in therapeutic trials as clinically and physiologically meaningful surrogate outcomes. Biomarkers data are used for accurate diagnosis and guidance of treatment in wide and varied medical conditions, but efforts to expand their use in pARDS continue to suffer from challenges due to the relatively low incidence of the disease, standardization of diagnostic criteria and measurement techniques, and funding limitations. Collaboration between nations and institutions is essential for replication of initial results, continued innovation, and to expand the applicability of findings to more diverse pediatric populations.

## Author Contributions

Drs. BO and AS conceived of the project and jointly prepared the abstract proposal. Dr. BO drafted the manuscript. Dr. AS provided subject matter guidance and critically reviewed and revised the manuscript. All authors approved of the final manuscript prior to publication.

## Conflict of Interest Statement

The authors declare that the research was conducted in the absence of any commercial or financial relationships that could be construed as a potential conflict of interest.

## Footnotes

^1^www.palisi.org

^2^www.cpccrn.org
